# Association between systemic inflammatory markers and recurrence risk in benign paroxysmal positional vertigo: a retrospective cohort study

**DOI:** 10.3389/abp.2026.16188

**Published:** 2026-03-23

**Authors:** Bekir Doğan, Deniz Baklacı

**Affiliations:** 1 Department of Otorhinolaryngology, Zonguldak Atatürk State Hospital, Zonguldak, Türkiye; 2 Department of Otorhinolaryngology, Faculty of Medicine, Zonguldak Bülent Ecevit University, Zonguldak, Türkiye

**Keywords:** benign paroxysmal positional vertigo (BPPV), CRP, neutrophil-to-lymphocyte ratio, recurrence, systemic immune-inflammation index (SII)

## Abstract

**Objective:**

Benign paroxysmal positional vertigo (BPPV) is a common vestibular disorder that responds well to canalith repositioning maneuvers but is frequently complicated by recurrence. Increasing evidence suggests that systemic inflammation may influence disease course and recurrence risk. This study aimed to investigate the association between routinely available systemic inflammatory markers and BPPV recurrence within 6 months.

**Methods:**

In this single-center retrospective cohort study, 300 adult patients diagnosed with BPPV between January 2020 and December 2024 were included. Neutrophil-to-lymphocyte ratio (NLR), systemic immune-inflammation index (SII), and C-reactive protein (CRP) levels measured at initial presentation were analyzed. Recurrence within 6 months following successful canalith repositioning maneuvers was defined as the primary outcome. Group comparisons were performed using non-parametric tests. Independent predictors of recurrence were identified using multivariable logistic regression analysis, and receiver operating characteristic (ROC) analysis was used to evaluate discriminatory performance.

**Results:**

During the six-month follow-up period, recurrence occurred in 78 patients (26.0%). Patients with recurrence had significantly higher NLR and CRP levels compared with those without recurrence (both *p* < 0.001). SII values were also elevated in the recurrence group, although the difference was less pronounced (*p* = 0.048). In multivariable analysis adjusted for age, sex, and canal involvement, both NLR (odds ratio [OR]: 1.42; 95% confidence interval [CI]: 1.18–1.71) and CRP (OR: 1.27; 95% CI: 1.10–1.46) emerged as independent predictors of BPPV recurrence. ROC analysis demonstrated moderate discriminatory ability for NLR (AUC = 0.71) and CRP (AUC = 0.69), whereas SII showed limited predictive performance (AUC = 0.62).

**Conclusion:**

Systemic inflammatory markers, particularly NLR and CRP, are independently associated with recurrence risk in BPPV. Given their low cost and widespread availability, these markers may provide complementary information for early risk stratification. Further prospective and multicenter studies are required to clarify causal mechanisms and confirm clinical applicability.

## Introduction

Benign paroxysmal positional vertigo (BPPV) is one of the most prevalent causes of peripheral vertigo and is characterized by brief episodes of positional vertigo accompanied by characteristic nystagmus. The accepted pathophysiological mechanism involves the detachment of otoconia from the utricular macula and their migration into the semicircular canals, resulting in abnormal endolymphatic flow and inappropriate activation of vestibular afferents (Bhattacharyya et al., 2017; von Brevern et al., 2015). Canalith repositioning maneuvers are highly effective in restoring normal vestibular function; however, recurrence remains a frequent and clinically relevant problem (Hilton and Pinder, 2014).

BPPV recurrence poses a significant challenge in long-term disease management and is associated with reduced quality of life, functional impairment, and increased healthcare utilization. Reported recurrence rates vary considerably among studies, with a substantial proportion of recurrences occurring within the first six months after successful treatment (Perez et al., 2012; von Brevern et al., 2007). This variability suggests that factors beyond the mechanical displacement of otoconia may contribute to disease persistence and recurrence.

Although BPPV has traditionally been considered a purely mechanical vestibular disorder, growing evidence indicates that systemic biological processes may influence its clinical course. In particular, systemic inflammation, microvascular dysfunction, and metabolic disturbances have been proposed to impair inner ear microcirculation and alter the biochemical stability of the otolithic membrane, thereby predisposing to recurrent otoconial detachment (Kim and Zee, 2014; Zahorec, 2001; Hu et al., 2014). These mechanisms highlight a potential link between systemic inflammatory status and vestibular pathology.

Inflammatory biomarkers derived from routine laboratory tests have gained increasing attention as indicators of systemic inflammatory activity. The neutrophil-to-lymphocyte ratio (NLR) and the systemic immune-inflammation index (SII) integrate information from different leukocyte subpopulations and have demonstrated prognostic value in a wide range of inflammatory, cardiovascular, and neurological conditions (Fest et al., 2020; Pepys and Hirschfield, 2003; Ishiyama et al., 2018). C-reactive protein (CRP), a well-established acute-phase reactant, remains a robust marker of systemic inflammation and is widely used in both clinical and research settings (De Stefano et al., 2014).

Despite emerging interest in the role of inflammation in vestibular disorders, data regarding the association between systemic inflammatory markers and BPPV recurrence are limited and inconsistent. Moreover, the independent predictive value of these markers after adjustment for relevant clinical variables has not been fully elucidated.

Therefore, the present study aimed to investigate the relationship between systemic inflammatory markers measured at initial presentation—namely NLR, SII, and CRP—and BPPV recurrence within 6 months. Additionally, we sought to evaluate the independent predictive value of these markers using multivariable models that account for key clinical confounders.

## Materials and methods

### Study design and ethical approval

This study was designed as a single-center retrospective cohort analysis. The study population comprised adult patients who presented to the otorhinolaryngology outpatient clinic and were diagnosed with BPPV between January 1, 2020, and December 31, 2024. Clinical and laboratory data were retrospectively extracted from the hospital’s electronic medical record system.

The diagnosis of BPPV was established based on a typical clinical history of vertigo triggered by changes in head position, together with the presence of characteristic positional nystagmus elicited during the Dix–Hallpike and/or supine roll tests. Consecutive patients who fulfilled the diagnostic criteria were included to minimize selection bias.

The study was approved by the Institutional Review Board at Zonguldak Bülent Ecevit University (Approval No2025/27) Owing to the retrospective design and the use of anonymized data, the requirement for informed consent was waived by the ethics committee. All procedures were conducted in accordance with the ethical standards of the Declaration of Helsinki.

#### Inclusion criteria

Patients were eligible for inclusion if they met all of the following criteria:

Age ≥18 years.

A confirmed diagnosis of BPPV based on a compatible clinical history and positive positional testing.

Availability of complete blood count parameters and CRP measurements obtained at initial presentation or within the first 24 h.

Availability of clinical follow-up data for a minimum of 6 months, or documentation of a repeat clinical evaluation within this period for assessment of BPPV recurrence.

#### Exclusion criteria

Patients were excluded from the analysis if any of the following conditions were present:

Neurological findings suggestive of central vertigo or radiological/clinical evidence of central nervous system pathology.

Coexisting vestibular migraine, Ménière’s disease, or other peripheral vestibular disorders.

Presence of active infection or clinically significant systemic inflammatory conditions at the time of presentation.

History of hematological malignancy or current use of immunosuppressive therapy.

Incomplete or missing essential clinical or laboratory data.

These exclusion criteria were applied to reduce potential confounding factors that could influence systemic inflammatory markers and to enable a more accurate assessment of their association with BPPV recurrence.

### Clinical and demographic data

Demographic variables, including age and sex, were obtained from patients’ medical records. The affected ear and the involved semicircular canal were determined based on findings from clinical examination and positional testing. Canal involvement was classified as posterior, lateral, anterior, or multicanal involvement.

The type of canalith repositioning maneuver performed for each patient was recorded according to the involved canal. These included the Epley maneuver for posterior canal BPPV, the barbecue roll maneuver for lateral canal involvement, and other appropriate maneuvers as clinically indicated.

### Laboratory assessment

All laboratory parameters were obtained from venous blood samples collected at the time of initial presentation. Neutrophil, lymphocyte, and platelet counts were retrieved from complete blood count analyses performed using standard automated hematology analyzers. Based on these parameters, the following systemic inflammatory markers were calculated:

Neutrophil-to-lymphocyte ratio (NLR): neutrophil count/lymphocyte count.

Systemic immune-inflammation index (SII): (neutrophil count × platelet count)/lymphocyte count.

CRP levels (mg/L), measured at the same presentation, were also included in the analysis. All laboratory analyses were conducted in the hospital’s central laboratory in accordance with standardized operating procedures.

### Outcome measures

The primary outcome was the occurrence of BPPV recurrence within 6 months following successful canalith repositioning maneuvers. Recurrence was defined as the reappearance of vertigo symptoms triggered by changes in head position, together with the re-identification of characteristic positional nystagmus on the Dix–Hallpike and/or supine roll tests.

The six-month follow-up interval was selected based on prior evidence indicating that BPPV recurrences most frequently occur during this period. Patients without documented repeat clinical visits or recorded symptoms suggestive of recurrence during the follow-up period were classified as having no recurrence.

### Statistical analysis

Statistical analyses were performed using IBM SPSS Statistics for Windows (Version 26.0; IBM Corp., Armonk, NY, USA). The distribution of continuous variables was evaluated using visual inspection and appropriate normality tests. Continuous variables with non-normal distribution were expressed as median and interquartile range (IQR), whereas categorical variables were summarized as counts and percentages.

Comparisons between patients with and without BPPV recurrence were conducted using the Mann–Whitney U test for continuous variables and the chi-square test or Fisher’s exact test, as appropriate, for categorical variables. All statistical tests were two-tailed, and a *p* value <0.05 was considered statistically significant.

To identify independent factors associated with BPPV recurrence, multivariable logistic regression analyses were performed. Age, sex, and canal involvement were included as covariates in all models. To account for potential collinearity among inflammatory markers, two separate multivariable models were constructed: a primary model including NLR and CRP, and an alternative model including SII and CRP.

Regression results were reported as odds ratios (ORs) with corresponding 95% confidence intervals (CIs). In a secondary analysis, the discriminatory performance of inflammatory markers for predicting BPPV recurrence was evaluated using receiver operating characteristic (ROC) curve analysis, with calculation of the area under the curve (AUC).

Additionally, to address potential misclassification of the primary outcome due to loss to follow-up, a sensitivity analysis was performed by restricting the study population to patients with at least one documented clinical follow-up visit within the six-month period.

## Results

### Patient characteristics

A total of 300 patients diagnosed with BPPV were included in the analysis. Of these, 170 patients (56.7%) were female and 130 (43.3%) were male. The median age of the study population was 58 years (interquartile range [IQR]: 49–67). Posterior semicircular canal involvement was the most common finding (225 patients, 75.0%), followed by lateral canal involvement (60 patients, 20.0%), anterior canal involvement (5 patients, 1.7%), and multicanal involvement (10 patients, 3.3%). Baseline demographic and clinical characteristics are summarized in Table 1.

**TABLE 1:** Demographic and clinical characteristics of patients with BPPV (n = 300).

Age, years, median (IQR)	58 (49–67)
Female sex, n (%)	170 (56.7)
Male sex, n (%)	130 (43.3)
Posterior semicircular canal involvement, n (%)	225 (75.0)
Lateral semicircular canal involvement, n (%)	60 (20.0)
Anterior semicircular canal involvement, n (%)	5 (1.7)
Multicanal involvement, n (%)	10 (3.3)

Data are presented as median (interquartile range, IQR) or number (percentage).

### Comparison of patients with and without recurrence

#### Demographic characteristics according to recurrence status

During the six-month follow-up period, BPPV recurrence was observed in 78 patients (26.0%). Recurrence rates did not differ significantly between female and male patients (26.5% vs. 25.4%, respectively; *p* = 0.82). Similarly, age distribution was comparable between patients with and without recurrence (*p* = 0.41). Detailed demographic comparisons between the two groups are presented in Table 2.

**TABLE 2:** Comparison of demographic characteristics between patients with and without BPPV recurrence.

Variable	No recurrence (n = 222)	Recurrence (n = 78)	p value
Age, years, median (IQR)	57 (48–66)	59 (50–68)	0.41
Female, n (%)	125 (56.3)	45 (57.7)	0.82
Male, n (%)	97 (43.7)	33 (42.3)	​

Percentages for sex are calculated within each recurrence-status group.

#### Inflammatory markers and BPPV recurrence

Patients who experienced recurrence exhibited significantly higher levels of NLR and CRP compared with those without recurrence. Median NLR was 2.9 (IQR: 2.2–3.6) in the recurrence group versus 2.1 (IQR: 1.6–2.7) in the non-recurrence group (*p* < 0.001). Median CRP levels were 2.9 mg/L (IQR: 1.9–4.1) and 1.8 mg/L (IQR: 1.1–2.6), respectively (*p* < 0.001). SII values were also higher in patients with recurrence, although the difference was less pronounced (median 640 [IQR: 480–820] vs. 560 [IQR: 410–710]; *p* = 0.048). These findings are summarized in Table 3.

**TABLE 3:** Comparison of systemic inflammatory markers according to BPPV recurrence status.

Marker	No recurrence (n = 222), median (IQR)	Recurrence (n = 78), median (IQR)	p value
NLR	2.1 (1.6–2.7)	2.9 (2.2–3.6)	<0.001
CRP, mg/L	1.8 (1.1–2.6)	2.9 (1.9–4.1)	<0.001
SII	560 (410–710)	640 (480–820)	0.048

NLR, neutrophil-to-lymphocyte ratio; SII, systemic immune-inflammation index; CRP, C-reactive protein.

#### Multivariable logistic regression analysis

Two multivariable logistic regression models adjusted for age, sex, and canal involvement (posterior versus lateral/other) were constructed to identify independent predictors of recurrence. In Model 1, both NLR (odds ratio [OR]: 1.42; 95% confidence interval [CI]: 1.18–1.71; *p* < 0.001) and CRP (OR: 1.27; 95% CI: 1.10–1.46; *p* = 0.002) were independently associated with BPPV recurrence. In Model 2, SII showed a statistically significant but modest association with recurrence. When scaled per 100-unit increase to improve clinical interpretability, SII was associated with an odds ratio of approximately 1.10, indicating an estimated 10.5% increase in the odds of recurrence for every 100-unit rise in SII (OR 1.105; 95% CI 1.000–1.221; p = 0.041), while CRP remained an independent predictor (OR: 1.25; 95% CI: 1.09–1.44; p = 0.003). The results of both models are detailed in Table 4.

**TABLE 4:** Multivariable logistic regression analysis of factors associated with BPPV recurrence.

Variable	Model 1 OR (95% CI)	p value	Model 2 OR (95% CI)	p value
Age (years)	1.02 (1.00–1.04)	0.048	1.01 (0.99–1.03)	0.11
Female sex	1.05 (0.63–1.76)	0.82	1.02 (0.61–1.71)	0.94
Posterior canal involvement	1.32 (0.74–2.34)	0.34	1.28 (0.72–2.29)	0.40
Neutrophil-to-lymphocyte ratio (NLR)	1.42 (1.18–1.71)	<0.001	—	—
Systemic immune-inflammation index (SII), (SII, per 100-unit increase)	—	—	1.105 (1.000–1.221)	0.041
C-reactive protein (CRP)	1.27 (1.10–1.46)	0.002	1.25 (1.09–1.44)	0.003

Two separate multivariable logistic regression models are presented. Model 1 includes NLR and CRP, whereas Model 2 includes SII and CRP. Both models are adjusted for age, sex, and canal involvement.

#### ROC analysis

Receiver operating characteristic (ROC) curve analysis demonstrated moderate discriminatory performance for NLR (area under the curve [AUC] = 0.71; 95% CI: 0.65–0.77) and CRP (AUC = 0.69; 95% CI: 0.63–0.75) in predicting BPPV recurrence. In contrast, SII exhibited more limited predictive ability (AUC = 0.62; 95% CI: 0.56–0.69). ROC analysis results are presented in Table 5.

**TABLE 5:** ROC analysis of NLR, CRP, and SII for predicting BPPV recurrence.

Marker	AUC	95% CI
NLR	0.71	0.65–0.77
CRP	0.69	0.63–0.75
SII	0.62	0.56–0.69

Receiver operating characteristic (ROC) analysis was applied to compare the discriminatory performance of NLR, CRP, and SII.

#### Sensitivity analysis

In the sensitivity analysis restricted to patients with at least one documented follow-up visit (n = 265), both NLR (OR: 1.39; 95% CI: 1.15–1.68; p < 0.001) and CRP (OR: 1.23; 95% CI: 1.07–1.42; p = 0.004) remained significantly associated with BPPV recurrence after adjustment for age, sex, and canal involvement. These findings were consistent with the primary analysis, supporting the robustness of the observed associations. Numerical results are detailed in Table 6.

**TABLE 6:** Sensitivity analysis of factors associated with BPPV recurrence.

Variable	OR	95% CI	p value
NLR	1.39	1.15–1.68	< 0.001
CRP	1.23	1.07–1.42	0.004
Age	1.01	0.98–1.05	0.472
Male sex	1.12	0.64–1.94	0.689
Lateral/other canal involvement	1.18	0.72–1.95	0.508

OR, odds ratio; CI, confidence interval.

Model adjusted for age, sex, and canal involvement.

## Discussion

In this retrospective cohort study, we investigated the association between systemic inflammatory markers measured at initial presentation and the risk of benign paroxysmal positional vertigo (BPPV) recurrence within 6 months. Our findings demonstrate that both the neutrophil-to-lymphocyte ratio (NLR) and C-reactive protein (CRP) were independently associated with recurrence after adjustment for key clinical variables, including age, sex, and canal involvement. Although the systemic immune-inflammation index (SII) was also statistically associated with recurrence, its predictive performance was comparatively limited. These results support a contributory role of systemic inflammation in BPPV recurrence and extend existing evidence by quantifying the independent predictive value of routinely available inflammatory biomarkers.

While the pathophysiology of BPPV has traditionally been attributed to mechanical displacement of otoconia, accumulating evidence suggests that systemic biological factors may also modulate disease susceptibility and recurrence. Impairment of vestibular microcirculation, inflammatory-mediated alterations in the biochemical stability of the otolithic membrane, and oxidative stress have been proposed as mechanisms facilitating recurrent otoconial detachment (Ishiyama et al., 2018; De Stefano et al., 2014). In this context, De Stefano et al. reported significant associations between BPPV recurrence and low-grade inflammation as well as metabolic factors, implicating inflammatory processes in recurrence pathogenesis (De Stefano et al., 2014). Similarly, Talaat et al. demonstrated elevated levels of low-grade inflammatory markers in patients with BPPV compared with healthy controls (Talaat et al., 2015). Recent systematic reviews and large cohort studies have identified multiple comorbidities influencing BPPV occurrence and recurrence, including metabolic, vascular, and inflammatory factors. For example, Alolayet and Murdin (2025) highlighted the role of systemic comorbidities such as hypertension, diabetes, and osteoporosis in BPPV pathogenesis and treatment outcomes (Alolayet and Murdin, 2025).

Conversely, Yang et al. reported increased systemic inflammatory markers in patients with BPPV compared with controls, although the relationship with recurrence was not comprehensively evaluated (Yang et al., 2018). Our study expands upon the existing literature by focusing specifically on recurrence as a clinically meaningful outcome and by applying multivariable regression models to control for relevant confounding factors. The persistence of NLR and CRP as independent predictors after adjustment underscores their potential relevance as surrogate indicators of systemic inflammatory burden influencing vestibular pathology.

The differential performance of inflammatory markers observed in this study warrants further consideration. NLR integrates information from innate and adaptive immune cell populations and has been shown to reflect both acute and chronic inflammatory states across a wide range of clinical conditions (Zahorec, 2001). CRP, a classical acute-phase reactant synthesized in response to proinflammatory cytokines, remains a robust and reliable biochemical marker of systemic inflammation (Pepys and Hirschfield, 2003). In contrast, SII incorporates platelet counts in addition to leukocyte parameters and has been primarily validated as a prognostic marker in oncological and cardiovascular diseases (Hu et al., 2014; Fest et al., 2020). Given the predominantly localized nature of the vestibular system, a relatively limited contribution of platelet-mediated mechanisms to BPPV recurrence may partly explain the weaker predictive performance of SII observed in our cohort, rendering these findings biologically plausible. To facilitate clinical interpretation, we expressed the association between SII and recurrence per 100-unit increase, which corresponded to an approximate 10.5% increase in the odds of recurrence for each 100-unit rise in SII. Recent clinical research has further elucidated the predictive value of inflammatory markers in BPPV recurrence. Liu et al. reviewed risk factors for BPPV recurrence, emphasizing the contribution of systemic inflammation and metabolic abnormalities (Liu et al., 2025). Additionally, Teggi et al. (2021) found that autoimmune and vascular factors, including inflammatory status, are associated with higher recurrence rates (Teggi et al., 2021). While the statistical association between CRP and BPPV recurrence is evident, the absolute difference reported in our study is modest and falls within the range of low-grade, nonspecific systemic inflammation. From a clinical standpoint, CRP is influenced by numerous confounding factors, including subclinical infections, metabolic disturbances, obesity, smoking status, and stress responses. Therefore, it is challenging to utilize CRP as a standalone or even adjunctive prognostic marker in routine outpatient practice due to this high background variability and the lack of a clear actionable threshold.

Heterogeneity among previous studies may be attributed to small sample sizes, short follow-up durations, and insufficient adjustment for confounding variables (De Stefano et al., 2014; Talaat et al., 2015; Alolayet and Murdin, 2025; Yang et al., 2018). In this regard, the relatively large sample size of the present cohort, the clinically relevant six-month follow-up period—during which most BPPV recurrences are known to occur—and the use of multivariable statistical models represent important methodological strengths that may enhance the robustness and generalizability of our findings. Furthermore, our sensitivity analysis restricted to patients with documented follow-up visits supported the robustness of the associations between systemic inflammatory markers and BPPV recurrence. Both NLR and CRP remained significant predictors in this subgroup, reinforcing the validity of our primary results despite potential misclassification bias from incomplete follow-up data. This methodological addition strengthens the confidence in the observed relationships and addresses concerns regarding under-ascertainment of recurrence.

From a clinical perspective, the observed association between recurrence risk and readily available, low-cost inflammatory markers such as NLR and CRP suggests a potential complementary role for these parameters in early risk stratification. Nevertheless, these biomarkers should not be interpreted as standalone diagnostic or prognostic tools but rather as adjuncts to clinical evaluation and vestibular examination findings (Ozdemir et al., 2019; Lippi et al., 2020).

Several limitations should be acknowledged. The retrospective design precludes causal inference, and inflammatory markers were assessed at a single time point, which does not account for temporal fluctuations in systemic inflammatory status. Prospective, multicenter studies incorporating longitudinal assessment of inflammatory markers are warranted to validate these findings and to further elucidate the mechanistic links between systemic inflammation and BPPV recurrence. The challenge of predicting recurrence remains central to BPPV management. Although the absolute difference in median CRP levels between recurrence groups was statistically significant and independent in multivariable models, its clinical relevance must be interpreted with caution. The observed values approach the lower detection limits of many standard laboratory assays, which may lack the sensitivity required to reliably discriminate within this low range. Consequently, while these findings suggest a biological link, the real-world clinical utility of conventional CRP at these concentrations remains limited without further validation using high-sensitivity assays (hs-CRP). ROC curve analyses provided cutoff values and performance metrics to aid clinical interpretation. Nonetheless, further studies are needed to establish standardized thresholds for clinical decision-making. Exclusion of patients with active infections or clinically significant systemic inflammatory conditions was intended to reduce confounding and improve internal validity. However, this approach may limit the generalizability of our findings to typical outpatient BPPV populations, where such comorbidities are common. Clinicians should consider this when applying our results, and future studies including broader patient populations are needed. The timing between symptom onset and laboratory assessment may influence inflammatory marker levels, as these biomarkers can vary dynamically during the disease course. Unfortunately, consistent data on symptom duration prior to presentation were not available in our retrospective cohort, limiting our ability to adjust for this factor. This limitation should be considered when interpreting the associations observed. Our study adjusted for key clinical variables such as age, sex, and canal involvement; however, data on other important recurrence-associated factors including metabolic diseases (e.g., diabetes mellitus), osteoporosis, vitamin D deficiency, head trauma, and migraine phenotype were not comprehensively available in our retrospective dataset. We acknowledge that elevated NLR and CRP levels may partly reflect these underlying systemic comorbidities rather than serving as fully independent prognostic markers. Consequently, in the absence of comprehensive comorbidity data, these markers should be viewed as indicators of a general inflammatory burden that may predispose to recurrence, rather than specific, standalone predictors. This conceptual clarification appropriately tempers the interpretation of these markers as independent predictors within our retrospective cohort. Future prospective studies incorporating these variables are warranted to clarify these relationships. Longitudinal studies have demonstrated that patients with comorbid autoimmune or vascular disorders, as well as those with persistent inflammatory markers, have increased risk of BPPV recurrence. Acle-Cervera et al. reported that despite symptom resolution, many patients experience recurrent vertigo and associated symptoms, underscoring the need for comprehensive management strategies addressing systemic inflammation (Acle-Cervera et al., 2025).

## Conclusion

In this retrospective cohort study, NLR and CRP levels measured at initial presentation demonstrated a statistical association with BPPV recurrence within a six-month follow-up period, even after adjustment for age, sex, and canal involvement. However, the absolute difference in CRP levels between groups was modest, and the discriminatory performance of these markers remained moderate. In contrast, while the SII was statistically associated with recurrence, its predictive performance was comparatively limited.

These findings suggest that systemic inflammatory processes may contribute to the clinical course of BPPV beyond purely mechanical factors. Nevertheless, given their limited specificity and the influence of various systemic confounders, NLR and CRP should not be viewed as standalone prognostic tools. Instead, they may serve as complementary indicators for recurrence risk assessment when interpreted cautiously alongside thorough clinical and vestibular evaluations. Furthermore, the modest absolute differences and the limitations of conventional assays highlight the need for further validation. Prospective, multicenter studies using high-sensitivity assays are warranted to establish standardized clinical thresholds and to determine the true clinical utility of incorporating these markers into risk stratification strategies.

## Data Availability

The raw data supporting the conclusions of this article will be made available by the authors, without undue reservation.
